# Palovarotene Can Attenuate Heterotopic Ossification Induced by Tendon Stem Cells by Downregulating the Synergistic Effects of Smad and NF-*κ*B Signaling Pathway following Stimulation of the Inflammatory Microenvironment

**DOI:** 10.1155/2022/1560943

**Published:** 2022-04-28

**Authors:** Junchao Huang, Jialiang Lin, Congbin Li, Bo Tang, Haijun Xiao

**Affiliations:** ^1^Department of Orthopedics, Anhui University of Science and Technology Affiliated Shanghai Fengxian Hospital, Shanghai 201400, China; ^2^Department of Orthopedics, Shanghai Fengxian District Central Hospital, Shanghai 201400, China

## Abstract

Heterotopic ossification (HO) is defined as the formation of bone tissues outside the bones, such as in the muscles. Currently, the mechanism of HO is still unclear. Tendon stem cells (TSCs) play important roles in the occurrence and development of HO. The inflammatory microenvironment dominated by macrophages also plays an important role in the course of HO. The commonly used clinical treatment methods, such as nonsteroidal anti-inflammatory drugs and radiotherapy, have relatively large side effects, and more efficient treatment methods are needed in clinical practice. Under physiological conditions, retinoic acid receptor (RAR) signal transduction pathway inhibits osteogenic progenitor cell aggregation and chondrocyte differentiation. We focus on palovarotene, a retinoic acid *γ*-receptor activator, showing an inhibitory effect on HO mice, but the specific mechanism is still unclear. This study was aimed at exploring the specific molecular mechanism of palovarotene by blocking osteogenic differentiation and HO formation of TSCs in vitro and in vivo in an inflammatory microenvironment. We constructed a coculture model of TCSs and polarized macrophages, as well as overexpression and knockdown models of the Smad signaling pathway of TCSs. In addition, a rat model of HO, which was constructed by Achilles tendon resection, was also established. These models explored the role of inflammatory microenvironment and Smad signaling pathways in the osteogenic differentiation of TSCs which lead to HO, as well as the reversal role played by palovarotene in this process. Our results suggest that, under the stimulation of inflammatory microenvironment and trauma, the injured site was in an inflammatory state, and macrophages were highly concentrated in the injured site. The expression of osteogenic and inflammation-related proteins, as well as Smad proteins, was upregulated. Osteogenic differentiation was performed in TCSs. We also found that TCSs activated Smad and NF-*κ*B signaling pathways, which initiated the formation of HO. Palovarotene inhibited the aggregation of osteogenic progenitor cells and macrophages and attenuated HO by blocking Smad and NF-*κ*B signaling pathways. Therefore, palovarotene may be a novel HO inhibitor, while other drugs or antibodies targeting Smad and NF-*κ*B signaling pathways may also prevent or treat HO. The expressions of Smad5, Id1, P65, and other proteins may predict HO formation.

## 1. Introduction

Heterotopic ossification (HO) is an abnormal growth of the laminar bone in soft tissues. Benign HOs have no evident symptoms; however, when the disease progresses, the activities of the adjacent joints decrease initially [1], but the pathogenesis is still unknown. Currently, the commonly used clinical treatment methods such as nonsteroidal anti-inflammatory drugs (NSAIDs) and radiotherapy have relatively remarkable side effects [2]. Thus, more efficient treatment methods are needed in clinical practice. Palovarotene is a retinoic acid gamma receptor (RAR-*γ*) activator, a member of the nuclear receptor superfamily, which is closely related to cartilage formation [3]. In the process of cartilage formation, a low concentration of endogenous retinoic acid is required, so the administration of retinoic acid receptor agonists may inhibit the formation of heterotopic ossification [4]. Studies have shown that palovarotene can inhibit the formation of heterotopic bone in HO model mice generated, but the exact mechanism is unknown [5]. Currently, progenitor cells, signaling pathway molecules, and the local microenvironment are considered the three main elements of HO formation, and new drugs that can interfere with the formation of these three elements may have the potential to prevent and treat HO [6].

Tendon stem cells (TSCs) are stem cells with biological functions similar to mesenchymal stem cells (MSCs). A study reported that TSCs undergo osteogenic and chondrogenic differentiation under inflammatory conditions, which ultimately leads to HO in the tendon site [7]. Considering the three main factors of HO formation, the inflammatory microenvironment may be related to HO formation. Studies have demonstrated that the inflammatory microenvironment can affect HO formation in local TSCs [8]. Therefore, investigating the levels of inflammatory factors may indicate the progression of HO. Macrophages are actively involved in the formation of the inflammatory microenvironment, and it can polarize in inflammatory diseases [9]. M1 macrophages are classically activated as proinflammatory cells and play a crucial role in the host's resistance to infection. Conversely, M2 macrophages are related to anti-inflammatory and tissue remodeling reactions, and they represent the two ends of macrophage activation. The transformation of macrophages with different phenotypes regulates the onset, progression, and termination of inflammatory diseases [10]. Signal transduction pathways related to osteogenesis mainly contributed to the transformation of osteogenic progenitor cells into the osteoblasts [11]. The activation of the Smad signal transduction pathway was observed in animal HO models, with the phosphorylation of Smad1 and Smad5 [12, 13].

Can palovarotene inhibit the Smad signaling pathway to deter HO formation? Our previous study also found that the NF-*κ*B signaling pathway is activated during HO [14]. Interestingly, Smad is also a downstream transcription molecule of the NF-*κ*B signaling pathway. While NF-*κ*B was inhibited, the mRNA level of Smad in the osteoblasts is also significantly reduced [15]. Do NF-*κ*B and Smad signaling pathways play a synergistic role in the course of HO? This study will explore the above questions.

## 2. Materials and Methods

### 2.1. Ethical Information

This study was approved by the Bioethics Committee of the Shanghai Sixth People's Hospital. Experiments involving animals were performed in strict accordance with relevant regulations. The number and suffering of the animals were minimized as much as possible.

### 2.2. Preparation of TSCs and Macrophages

Male SD rats (4 weeks, weight 200 ± 5 grams) were sacrificed with their necks cut off; rats were soaked in 75% alcohol for 5 min, then moved to the ultraclean workbench, and fixed in supine position. Cut the skin of the leg, take out the tendon on both sides of the thigh, carefully remove the tendon sheath and tendon tissue, and then cut the tendon into 1 mm^3^ small tissue. The digestive liquid was added to the tissue, then the suction was blown and mixed, and the digestion was performed with a 37°C constant temperature water bath and shaking table for 1 h. After digestion, the tissue was blown by suction for 20 times, filtered through a 200-mesh cell sieve; filtrate was collected and centrifuged at 300G for 5 minutes. The supernatant was discarded, and 5 mL of DMEM-L was added. The single cell suspension was adjusted to a cell density of 1 × 106/mL and was inoculated. The cell culture flask was placed in an incubator at 37°C and 5% CO2 for culture. The solution was changed after 48 h and subsequently after every 3 days to remove the cells attached to the wall so as to purify TSCs. When the confluency of cell growth reached 80%, the cells were digested with trypsin for passage. Subsequently, TSCs were identified by immunofluorescence (see Immunofluorescence for the specific steps).

Two 4-week-old male SD rats were soaked in 75% alcohol for about 30 min to obtain the femur and tibia of the rat, and then, the bone marrow was washed out with *α*MEM. The red blood cells were lysed with schizo erythrocyte solution. After the lysed, red blood cells were treated with *α*MEM complete medium containing MCSF (10 ng/mL) for intervention. After 5 days of culture, M0 macrophages from mice were obtained and then amplified to 2 × 107. They were placed into 6-well plates and became overgrown 24 hours later. Subsequently, DMEM without serum was used for 2 hours, and LPS equipped with 100 ng/mL was used for intervention to induce differentiation. After differentiation, immunofluorescence was used for identification. According to the results of immunofluorescence, CCR7 content proportion was more than 80%, which could be used as seed cells for intervention.

### 2.3. CCK8 Cell Proliferation Assay

Palovarotene was prepared with complete medium to act on TSCs with three concentration gradients of 0.5, 1, and 2 *μ*M. CCK-8 (Dojindo, #CK04) was added to each well at 24, 48, and 72 h, and the cells were cultured at 37°C in a 5% CO_2_ incubator for 3 h in dark. The optical density (OD) values were measured at 450 nm at the same time points with a microplate analyzer, and the influence of the measured OD values on cell proliferation was analyzed.

### 2.4. Establishment of a Coculture System between Macrophage Cell Line and Mouse TSCs

The macrophages of M1 were cultured in Corning Transwell TM cells, and TSCs were lined in the pore plate. Subsequently, the cells were combined with the pores in the pore plate to construct the coculture system of activated macrophages and TSCs. Osteogenic induction medium were used in coculture system to inducted osteogenesis. The osteogenic induction medium used in this study consisted of 10 nM dexamethasone, 50 *μ*g/mL ascorbic acid, 10 mM B-GP disodium, 10% fetal bovine serum, and high-glucose Dulbecco's Modified Eagle Medium (DMEM) (Gibco, MA, USA).

### 2.5. Protein Extraction and Western Blot Detection

After 14 days of coculture between macrophage and TSCs through osteogenic induction culture, the cells in each group were collected and washed with PBS; 1 mL of RIPA lysate (Biosharp, #BL504) with PMSF was added, and the cells were gently suspended and placed in ice bath for 30 min and were mixed every 15 min. The cells were centrifuged at 12,000 RPM at 4°C for 20 min. The supernatant was carefully transferred into a new Eppendorf tube, and the protein content was measured with a BCA kit (BioSharp, #BL521). The separation glue and 5% concentrate glue were prepared using the formula. The samples were then loaded according to the results of protein quantification. Electrophoresis was performed subsequently, with concentrated glue at 80 V for 20 min and with separation glue at 120 V for 1 h. When the dye reached the end of the gel, the power supply was cut off, and electrophoresis was stopped. The proteins in the film were then transferred to a polyvinylidene difluoride (PVDF) membrane, using 5% skimmed milk powder at room temperature for 1 h. OCN (Servicebio, #GB11233) or RUNX2 (Bioss, #bs1134R) or SOX9 (Bioss, #bs4177R), beta actin (Servicebio, #GB11001) or P65 (Abcam, #ab179463) or Smad1/5 (Abcam, #ab38449) or Id1 (Proteintech, #18475-1--AP) or p-Smad1/5 (Abcam, #ab51451) were added to the blocking solution to dilute to the desired concentration and were incubated overnight with the membrane at 4°C. The secondary antibody was then incubated, and the membrane of the primary antibody was washed 5 times with TBST, 10 min each time. According to the dosage, the secondary antibody corresponding to the primary antibody was diluted at 1 : 2000 and incubated together with the membrane at 37°C for 1 h. It was washed with TBST for 5 times, 10 min each. Lastly, images were taken using ECL (BioSharp, #BL520), an all-in-one chemiluminescence instrument. All the original full gels of Western blots are seen in Supplementary Figures 1.

### 2.6. Alizarin Red and Alkaline Phosphatase Staining

After 14 days of osteogenesis, alkaline phosphatase (ALP) staining was performed; 40 *μ*L of reagent A was added to 1 mL of reaction buffer, and 40 *μ*L of reagent B was added to the mix; that is, the reaction working solution (ready for use) was prepared (the reagents were purchased from Beyotime, #C3206). Subsequently, 1 mL of PBS was added to each well and was removed after 1 min, and the cells were washed twice. Subsequently, 500 *μ*L of fixing solution was added to each well, and the cells were fixed at 37°C (or room temperature) for 30 min. The reaction solution prepared was added to each well, and the cells were dyed at 37°C (or room temperature) for 30 min. Lastly, 1 mL of washing solution was added to each well; the cells were washed twice and observed under a microscope.

Then, after 21 days of osteogenesis, alizarin red staining (ARS) was performed as follows: 1 mL of PBS was added to each well and was removed after 1 min. Furthermore, 1 mL of 70% ethanol or 10% neutral formaldehyde was added to each well, and the cells were fixed at 37°C (or room temperature) for 30 min. The fixed solution was discarded, and 1 mL of washing solution was added to each well. The cells were washed thrice, and 3 mL of alizarin red S staining solution (purchased from Sigma, #A5533) was added to each well; the cells were stained at 37°C (or room temperature) for 15–20 min. The cells were washed twice with the washing solution and were observed under a microscope.

### 2.7. RT-PCR

The transcription level of osteogenic genes was detected by reverse transcription PCR (RT-PCR). The reaction system used was SYBR Green Mix (Takara, RR420A), and the fluorescence signal was obtained by a detecting instrument (Roche, Light Cycler 480). The primer sequences used in this study are shown in Table 1.

### 2.8. Immunofluorescence

TSCs, cocultured with macrophage, were digested and collected for cell slit. Palovarotene or LDN193189 and induction treatment were given according to the experimental group for 30 min. After washing the cells with PBS, 4% paraformaldehyde (Biosharp, # BL539A) was added, and the cells were fixed after 20 min at room temperature. PBS washes were given twice again, and 0.1% Tx-100 (Beyotime, # P0096) was added. After 15 min at room temperature, the liquid turned transparent, and 5% BSA/PBS was added, and the cells were incubated at 37°C for 1 h. The TPPP3 (Biorbyt, #5-F10) or CCR7 (Abcam, # ab32527) or p65 (Abcam, # ab16502) or Smad5 (Affinity, #AF5119) or Id1 (Proteintech, #18475-1--AP) antibody working fluid was added, and the cells were incubated at 4°C overnight. After overnight incubation, the samples were washed 3 times in PBS and were incubated at 37°C for 1 h with anti-rab-CY3 (ABCAM, #AB6939) working solution. The samples were washed 3 times in PBS and were incubated at room temperature for 15 min with Hoechst (ABCAM, #AB228551) and were washed 3 times in PBS. Lastly, the samples were seeded and photographed under a fluorescence microscope.

### 2.9. Micro-CT

Micro-CT analysis was performed after 12 weeks of feeding. Achilles tendons with lower tibia and calcaneus from mice were fixed in 10% formalin overnight. The X-ray tube settings were 50 kV and 60 *μ*A, and the images were acquired at 50 *μ*m resolution. A 0.5 rotation step through a 360 angular range with a 50 ms exposure per step was used. The images were reconstructed and analyzed with Skysan 1275 software.

### 2.10. Construction of HO Animal Model

The experimental rats were 24 male SD rats (4 weeks, weight 200 ± 5 grams), 8 in each group and 3 groups in total according to our experimental requirements. Rats purchased from Shanghai Jiesjie Experimental Animal Co., Ltd. First, the rats were given isoflurane (model: R510-22); after the general anesthesia took effect, the Achilles tendon area was disinfected with alcohol; sterile tissue was spread; 1 cm incision was cut along the Achilles tendon with a blade; and the subcutaneous tissue was separated layer by layer to expose the Achilles tendon, and blunt separation was performed to avoid artery injury. In all rats except the sham group, the Achilles tendon was cut off in the middle without suture after clamped for 8 times, and 5 mg amoxicillin was given to the wound. Finally, close the incision layer by layer with 4-0 silk thread, taking care not to suture to the Achilles tendon. In the sham group, the Achilles tendon was only exposed and then sutured. Finally, the surviving and healthy rats in each group were selected for follow-up experiments. All rats were fed for 12 weeks and treated for experiment.

### 2.11. Drug Preparation and Administration

Palovarotene was dissolved in dimethyl sulfoxide (DMSO) solvent, and 100 *μ*L of an oral solution was obtained by mixing 30 *μ*L of the drug solution and 70 *μ*L of corn oil. The sham group and vehicle group received the same proportion of DMSO and corn oil solution, and the concentration of palovarotene was 1 mg/kg/day. Palovarotene was administered orally with no. 20 gavage needle for 21 days.

LDN-193189 and Smad signaling pathway inhibitor were purchased from MCE, # HY-12071, diluted to 10 *μ*M.

### 2.12. HE, Safranin O-Fast Green Staining and Immunohistochemistry

Achilles tendon specimens were fixed in 10% formaldehyde solution for 1 day and then decalcified in 15% ethylenediamine tetraacetic acid (EDTA) for 2-3 weeks at 4°C. Then, it was dehydrated with anhydrous ethanol, embedded in paraffin, cut into 5 *μ*m thick slices with slicer, and put on slides. HE (Servebio, #G1005-1, G1005-2) and Safranin O-Fast Green (Solarbio, #G1371) staining and immunohistochemical staining were performed; P65 (Proteintech, #10745-1-AP) and OCN (Servebio, #GB11233) and Sox9 (Bioss, #4177R) and p-Smad1 (Abcam, #ab51451) and TNF-*α* (Affinity, #AF7014) and TGF-*β* (Abcam, #ab215725) and MMP-9 (Abcam, #ab283575) and IL-1*β* (Abcam, #ab9722) and IFN-*γ* (Affinity, #DF6045) and Id1 (Proteintech, #18475-1--AP) antibody concentrations were diluted 1 : 1000.

### 2.13. Transfection

The Smad5-pcDNA3.1 and siSmad5 were transfected into TSCs at a final concentration of 100 nM by Lipofectamine 2000 reagent (Invitrogen, Carlsbad, CA, USA). Transfected for 48 h, the cells were harvested for later experiments.

### 2.14. Statistical

Independent samples *t* test was used to confirm comparisons of the variables of 2 groups. One-way analysis of variance (ANOVA) was used to confirm comparisons of the variables of 4 groups. Multiple comparisons were performed by one-way ANOVA test followed by post hoc contrasts performed by least significant difference (LSD) test (if the ANOVA results were significant). *p* < 0.05 was considered statistically significant.

## 3. Results

### 3.1. TSCs Were Found in Rat's Achilles Tendon Tissue; TSCs and Macrophages from Rats Were Extracted and Identified

**Figure 1:**
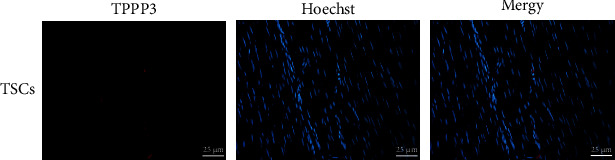
TSCs existed in tendon tissue were confirmed (*n* = 3). Scale bar: 25 *μ*m.

We selected healthy male SD rats (4 weeks, weight 200 ± 5 grams). Immunofluorescence was performed on the sections of its Achilles tendon tissue. We characterized TSCs with the surface-specific marker TPPP3+ [16] and characterized M1 macrophages with CCR7 [17]. The results showed that there were TSCs in the Achilles tendon tissue of rats (Figure 1). Next, TSCs and macrophages of the above-mentioned SD rat were extracted. The identity was confirmed by immunofluorescence.

### 3.2. The Inflammatory Microenvironment Promotes Osteogenic Differentiation of TSCs, and Palovarotene Can Influence Osteogenesis

**Figure 2:**
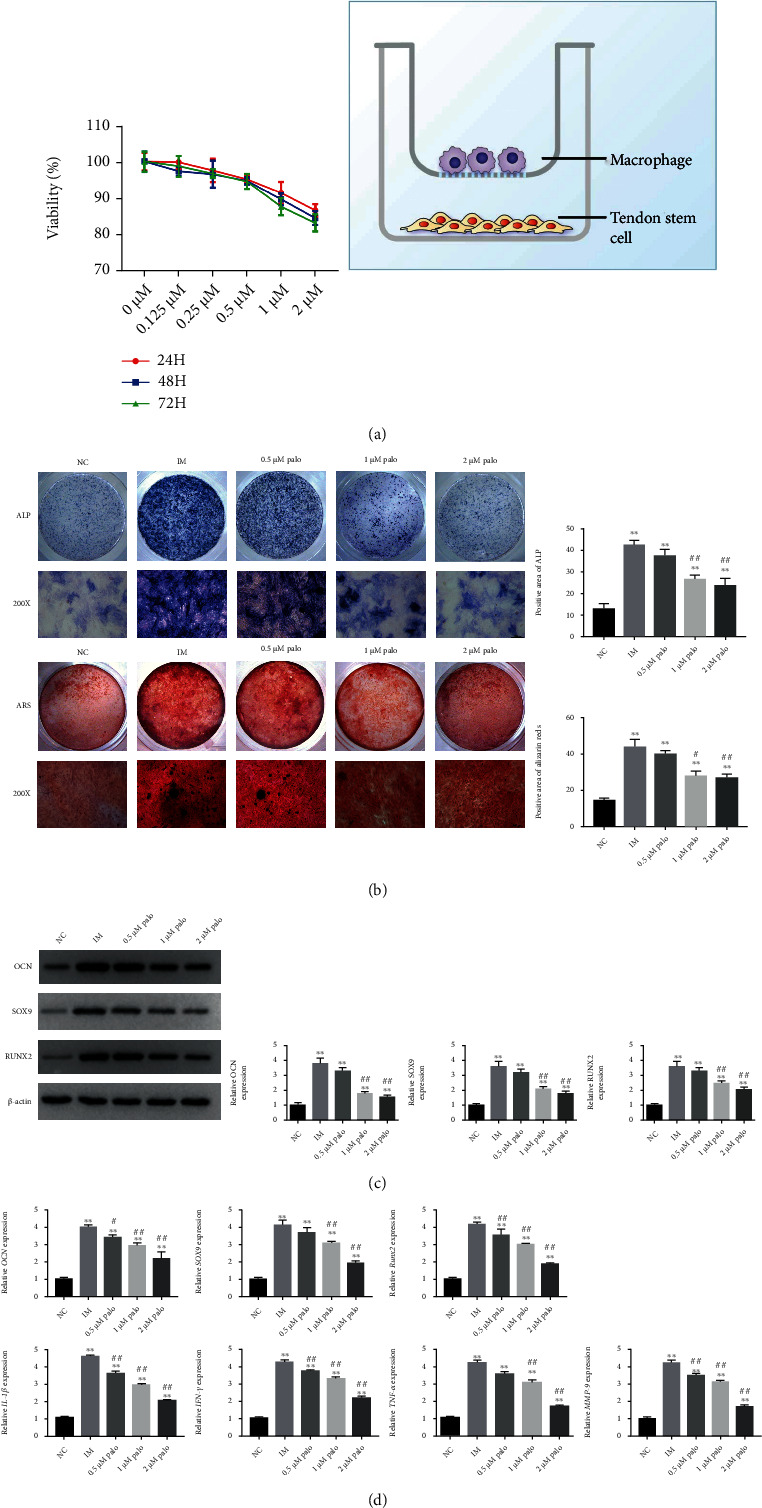
The inflammatory microenvironment promotes osteogenic differentiation of TSCs, and palovarotene can influence osteogenesis. (a) CCK8 proliferation assay was used to determine the dose range of palovarotene. Palovarotene at 0.5, 1, and 2 *μ*M concentration had less effect on TSCs proliferation. Coculture system of activated macrophages and TSCs was constructed. (b) ALP and ARS staining in TSCs after coculture with macrophages. Data are means ± SD (*n* = 3). ^∗^*p* < 0.05 and ^∗∗^*p* < 0.01. ^#^*p* < 0.05 and ^##^*p* < 0.01. Scale bar = 50 *μ*m. (c) Western blot analysis and relevant quantitative analysis of OCN, SOX9, and RUNX2. Data are means ± SD (*n* = 3). ^∗^*p* < 0.05 and ^∗∗^*p* < 0.01. ^#^*p* < 0.05 and ^##^*p* < 0.01. (d) The mRNA expression of OCN, SOX9, RUNX2, MMP-9, IL-1*β*, IFN-*γ*, and TNF-*α*. Data are means ± SD (*n* = 3). ^∗^*p* < 0.05 and ^∗∗^*p* < 0.01. ^#^*p* < 0.05 and ##*p* < 0.01.

To validate the effects of the inflammatory microenvironment on TSC osteogenic differentiation, we performed in vitro experiments in a control group (no load, NC group), inflammatory microenvironment (IM) group, and inflammatory microenvironment group plus palovarotene group (Palo group). First, we constructed a coculture system of activated macrophages and TSCs to simulate the inflammatory microenvironment in vitro. Second, we used Cell Counting Kit 8 (CCK8) proliferation assay to explore the suitable administration range of palovarotene, which was determined as 0.5 *μ*M, 1 *μ*M, and 2 *μ*M concentration gradient after screening (Figure 2(a)). Alizarin red and alkaline phosphatase staining showed that calcium deposits and alkaline phosphatase levels were significantly upregulated in the IM group compared with the NC group, which could be inhibited by palovarotene, and the inhibition degree increased with the increase of palovarotene concentration (Figure 2(b)). WB and RT-PCR results showed that compared with the NC group and the palovarotene group, the expression levels of osteogenic markers such as osteocalcin (OCN), runt-related transcription factor 2 (RUNX2), and sry-related HMG box-9 (SOX9) and inflammatory markers such as TNF-*α*, IL-1*β*, MMP9, TGF-*β*, and IFN-*γ* in the IM group were significantly increased. Palovarotene can reversed this trend (Figures 2(c) and 2(d)). The results suggested that the osteogenic differentiation of TSCs was enhanced when stimulated by inflammatory microenvironment, while palovarotene could inhibit this trends.

### 3.3. Osteogenic Differentiation Induced by Inflammatory Microenvironment Was Related to Smad Signaling Pathway

**Figure 3:**
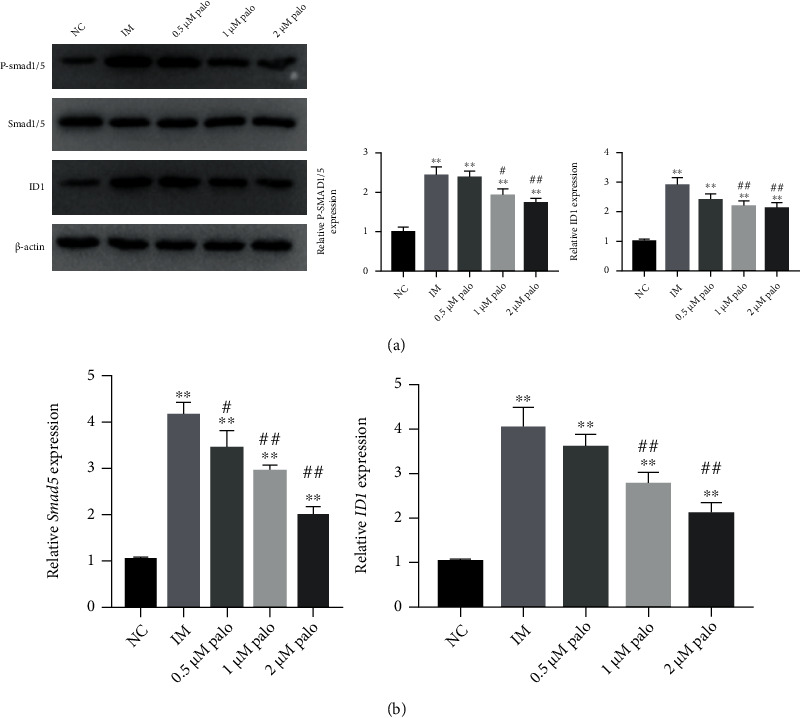
Smad signaling pathway was related to inflammatory microenvironment. (a) Western blot analysis and relevant quantitative analysis of P-Smad1/5, Smad1/5, and ID1 in TSCs. Data are means ± SD (*n* = 3). ^∗^*p* < 0.05 and ^∗∗^*p* < 0.01. ^#^*p* < 0.05 and ^##^*p* < 0.01. mRNA expression analysis of Smad5 and ID1. Data are means ± SD (*n* = 3). ^∗^*p* < 0.05 and ^∗∗^*p* < 0.01. ^#^*p* < 0.05 and ^##^*p* < 0.01.

We continued to find that, in our in vitro experiment, the WB and RT-PCR results showed that compared with the NC group and the palovarotene group, the expression levels of pSmad1/5, SMAD5, and ID1 in the IM group were significantly increased (Figures 3(a) and 3(b)). This revealed that when TSCs activated the Smad signaling pathway during stimulation by inflammatory microenvironment, pSMAD1/5, Smad5, and ID1 are downstream transcription molecules of Smad signaling pathway. This results suggested that Smad signaling pathway may play an important role in the pathological process of HO. Thus, more investigation are needed to be done.

### 3.4. Smad and NF-*κ*B Signaling Pathways Play a Synergistic Role in the Pathological Process of HO, and Palovarotene Acts as an Inhibitor of Smad Signaling Pathway Similar to LDN193189, Inhibited HO by the Inactivation of Smad and NF-*κ*B Signaling Pathways

**Figure 4:**
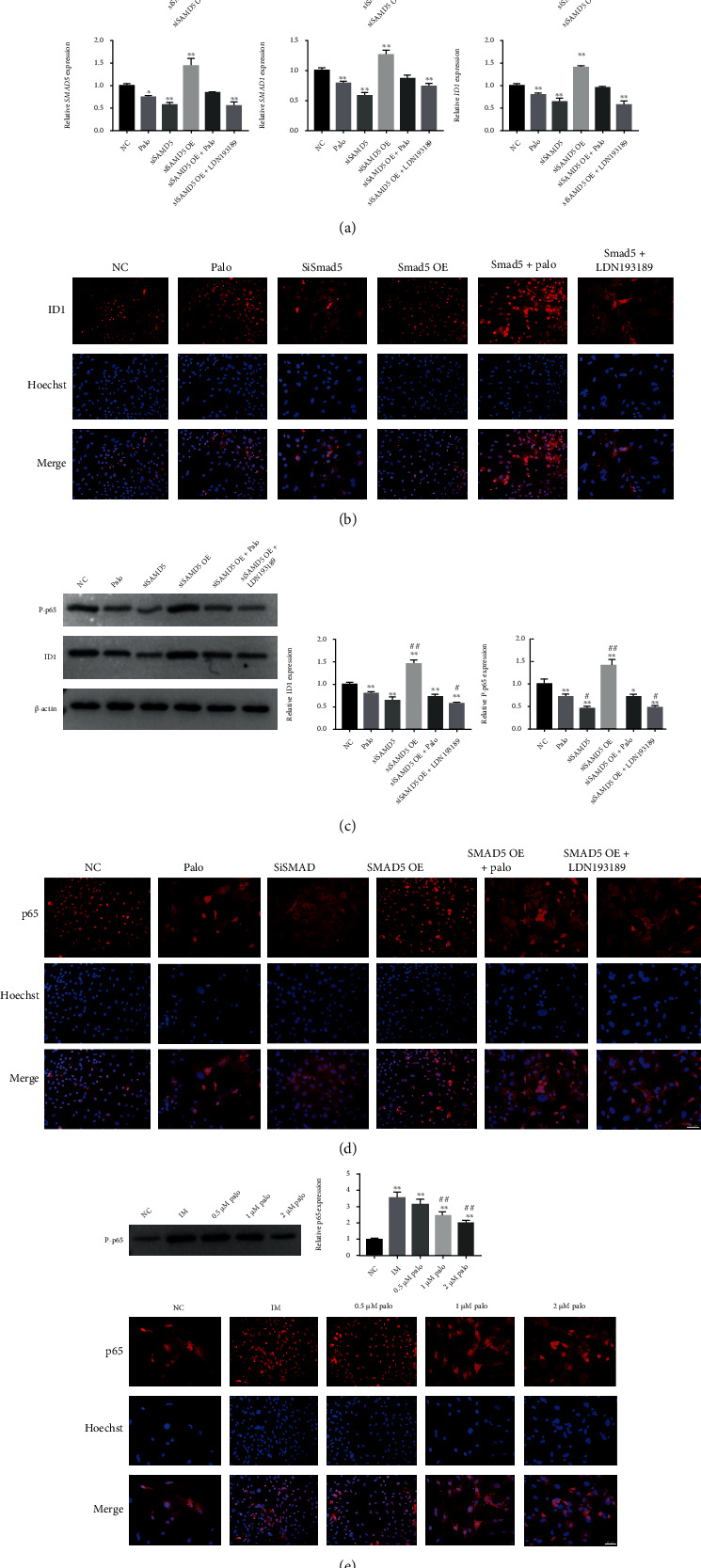
Smad and NF-*κ*B signaling pathways play a synergistic role in HO formation, and palovarotene inhibits HO formation by blocking the Smad and NF-*κ*B signaling pathways. (a) The mRNA expression of OCN, SOX9, RUNX2, Smad1, Smad5, and ID1. Data are means ± SD (*n* = 3). ^∗^*p* < 0.05 and ^∗∗^*p* < 0.01. ^#^*p* < 0.05 and ^##^*p* < 0.01. (b) Immunofluorescence against ID1 (*n* = 3). Scale bar = 25 *μ*m. (c) Western blot analysis and relevant quantitative analysis of p-p65 and ID1. Data are means ± SD (*n* = 3) ^∗^*p* < 0.05 and ^∗∗^*p* < 0.01. ^#^*p* < 0.05 and ^##^*p* < 0.01. (d) Immunofluorescence against p65 (*n* = 3). Scale bar = 25 *μ*m. (e) Western blot analysis and relevant quantitative analysis of p65 and immunofluorescence against P65 in the previous coculture model series experiments (*n* = 3). Data are means ± SD (*n* = 3). ^∗^*p* < 0.05 and ^∗∗^*p* < 0.01. ^#^*p* < 0.05 and ^##^*p* < 0.01. Scale bar = 25 *μ*m.

To further validate the synergistic effects of Smad and NF-*κ*B signaling pathways, in the pathological process of HO. We constructed a Smad signaling pathway overexpression and knockdown cell model of TSCs. After confirming the validity of the overexpression and knockdown models, we performed a series of cellular experiments, given palovarotene or not, given Smad signaling pathway inhibitor LDN193189 or Smad overexpressed, knockdown, or no-load NC. RT-PCR and immunofluorescence results showed that compared with NC alone, palovarotene and Smad5 knockdown plasmids downregulated the transcription levels of osteogenic and inflammatory markers, inactivated the Smad signaling pathway, and downregulated the Smad signaling pathway downstream transcription molecule Id1. Id1 was scattered throughout the cytoplasm. The administration of Smad5-overexpressed plasmid significantly upregulated the transcription levels of osteogenic and inflammatory markers, activated the Smad signaling pathway, and upregulated the downstream transcription molecule Id1. Id1 was activated and entered the nucleus. On this basis, the administration of palovarotene or the Smad signaling pathway inhibitor LDN193189 reversed this trend (Figures 4(a) and 4(b)). These results confirm our suspicion that palovarotene inhibits HO formation by inactivating the Smad signaling pathway. Interestingly, the protein levels of the key molecule P65 in the NF-*κ*B signaling pathway and ID1 were significantly increased. P65 were activated and entered the nucleus in the SMAD5-overexpressed plasmid group. These trends were reversed after palovarotene administration (Figures 4(c) and 4(d)). This suggests that not only the Smad signaling pathway but also the NF-*κ*B signaling pathway is involved in HO pathogenesis, which can be blocked by palovarotene. To verify this, we observed in the aforementioned in vitro experiment, WB results showed in the IM group, WB results revealed increased P65 levels, and immunofluorescence revealed that P65 moved from the cytoplasm into the nucleus to activate downstream pathways, which were also reversed by palovarotene (Figure 4(e)). These results suggest that Smad and NF-*κ*B signaling pathways play a synergistic role in HO formation, and palovarotene inhibits HO formation by blocking the Smad and NF-*κ*B signaling pathways.

### 3.5. Palovarotene Can Reduce the Formation of Ectopic Bones and the Accumulation of Macrophages in HO Model Rats

**Figure 5:**
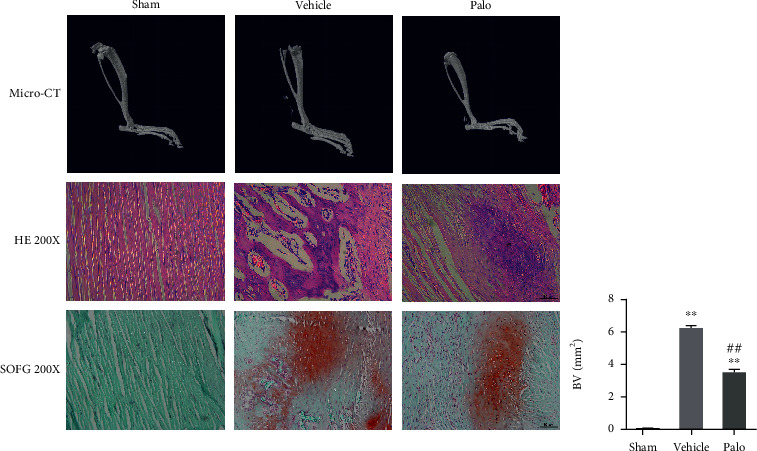
Micro-CT, HE, and SOFG staining revealed that palovarotene can attenuate HO. Data are means ± SD (*n* = 8). ^∗^*p* < 0.05 and ^∗∗^*p* < 0.01. ^#^*p* < 0.05 and ^##^*p* < 0.01. Scale bar = 50 *μ*m.

In a series of in vitro experiments, we confirmed that Smad and NF-*κ*B signaling pathways play a synergistic role in HO formation, and palovarotene inhibits HO formation by blocking the Smad and NF-*κ*B signaling pathways. Next, we verified this viewpoint through a series of in vivo experiment. We constructed the Achilles tendon HO model in rats, and the animals were divided into the sham group (sham group), Achilles tendon HO group (vehicle group), and Achilles tendon HO plus administration of palovarotene group (Palo group). The dose of palovarotene was 1 mg/kg/day, and the concentration was determined by the preliminary experimental results and relevant literature [18]. Twelve weeks after the rats were modeled, we started a series of experiments. Micro-CT findings revealed that compared with the sham group, HO formation was significantly increased in the vehicle group, while it was significantly inhibited in the Palo group. In the hematoxylin and eosin staining and FerroOrange/rapid green staining of rats in each group, the vehicle group demonstrated a disordered arrangement of Achilles tendon tissues, with multiple ectopic bone tissues, vascular ingrown, and macrophage aggregation. Conversely, the sham group and Palo group had an orderly arrangement of Achilles tendon fibers, with only a small amount of cartilage and ectopic bone formation (Figure 5).

### 3.6. Construction of HO Model Activated the Smad and NF-*κ*B Signaling Pathways, Increased Inflammatory Response, and Enhanced the Osteogenic Differentiation of Progenitor Cells, which Process Can Be Mitigated by Palovarotene

**Figure 6:**
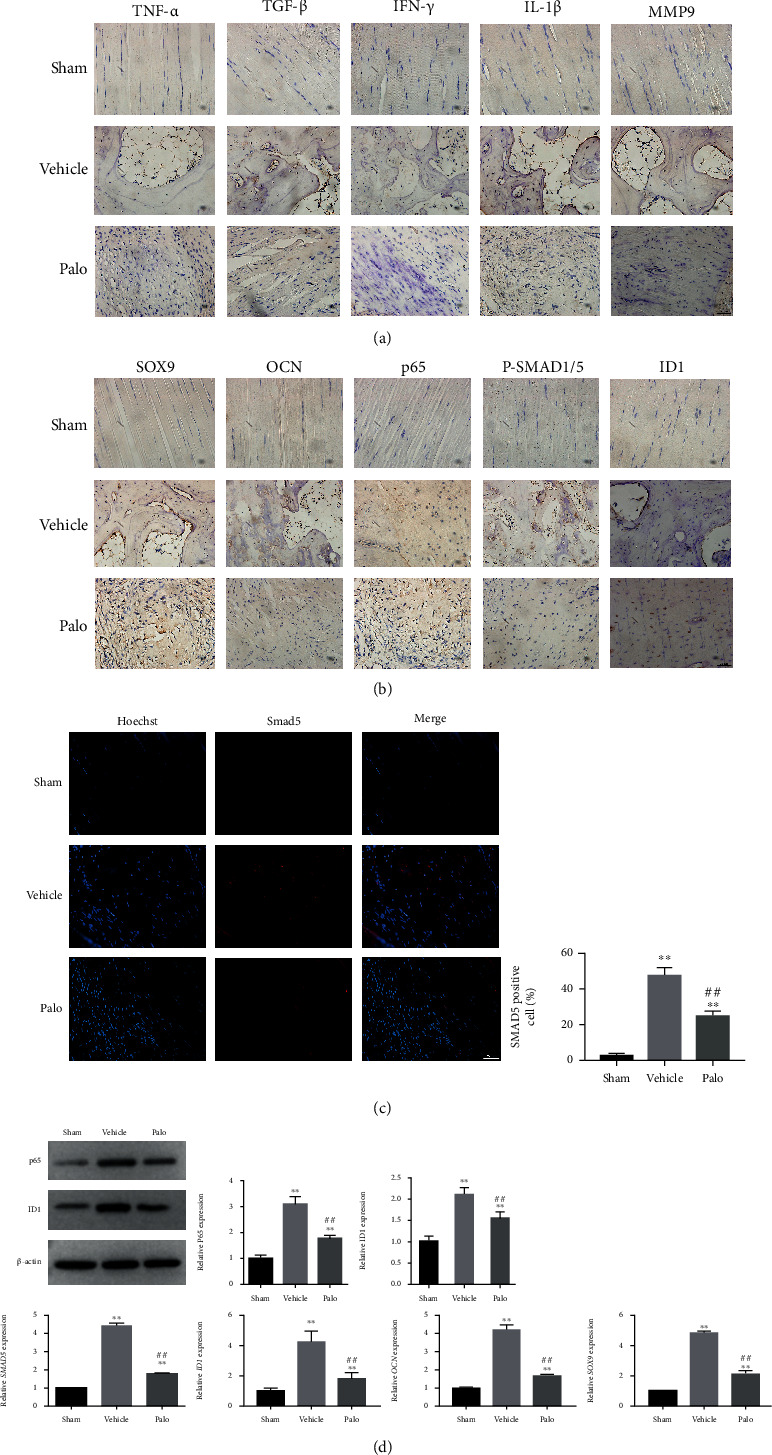
In vitro experimental further verifies the effects of palovarotene and Smad and NF-*κ*B signaling pathways in HO. In immunohistochemistry, the positive rate of inflammation such as TNF-*α*, TGF-*β*, IFN-*γ*, IL-1*β*, and MMP-9 was increased (a), as well as p65, ID1, p-SMAD1/5, and osteogenic genes, concluding OCN and SOX9 (b). Scale bar = 25 *μ*m. (c) Immunofluorescence and quantitative analysis against Smad5. Scale bar = 25 *μ*m. Data are means ± SD (*n* = 3) ^∗^*p* < 0.05 and ^∗∗^*p* < 0.01. ^#^*p* < 0.05 and ^##^*p* < 0.01. (d) The mRNA expression, Western blot analysis of tendon tissues, and relevant quantitative analysis of p65, ID1, OCN, and SOX9. Data are means ± SD (*n* = 3). ^∗^*p* < 0.05 and ^∗∗^*p* < 0.01. ^#^*p* < 0.05 and ^##^*p* < 0.01.

**Figure 7:**
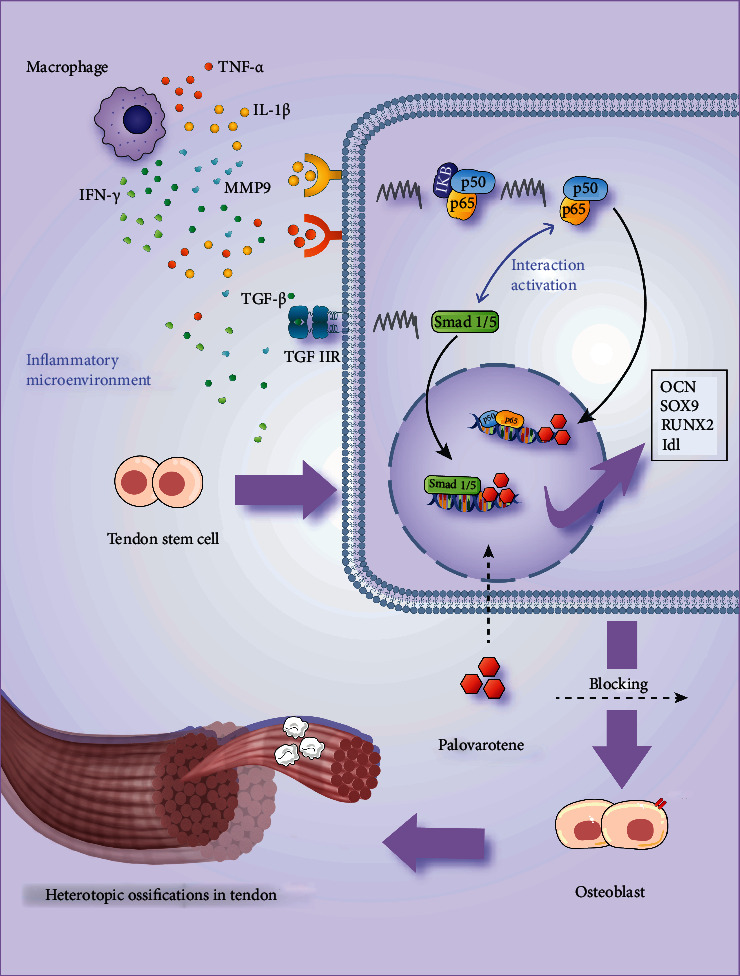
A demonstration of palovarotene's effects and the synergistic effects of Smad and NF-*κ*B signaling pathways following stimulation of the inflammatory microenvironment.

Immunohistochemical results in animal experiments revealed that inflammatory markers such as tumor necrosis factor (TNF)-*α*, interleukin (IL)-1*β*, matrix metallopeptidase 9 (MMP9), tumor growth factor (TGF)-*β*, and interferon (IFN)-*γ* were upregulated in the vehicle group (Figure 6(a)), while osteogenesis and chondrogenic markers OCN and SOX9 were upregulated; PSMAD1/5, a key molecule of the Smad signaling pathway, and Id1, a downstream transcription molecule, are also upregulated in the vehicle group (Figure 6(b)). Immunofluorescence shows that Smad5 in the vehicle group enters the nucleus and executes its function (Figure 6(c)). Western blot (WB) and quantitative polymerase chain reaction (RT-PCR) results after histone protein and RNA extraction from the rats showed increased transcription levels of OCN, SOX9, PSMAD1/5, SMAD5, and ID1, which was consistent with the immunohistochemical results and increased protein level of ID1 (Figure 6(d)). The results of the Palo group showed the same trend as that of the sham group; i.e., the upregulation of the osteogenesis and inflammatory markers was inhibited, the Smad signaling pathway was inactivated, and Smad5 was scattered in the cytoplasm. The above in vitro experimental results were consistent with our in vitro experiments and verified the conclusions that TSCs activate the Smad signaling pathway and enhance osteogenic differentiation to induce HO under inflammatory microenvironment and trauma stimulation. The NF-*κ*B signaling pathway acts synergistically in this process. Palovarotene may act on the common downstream transcription target of Smad and NF-*κ*B. Palovarotene inhibits HO by blocking Smad and NF-*κ*B signaling pathways (Figure 7).

## 4. Discussion

HO can be divided into two types according to its causes, namely, genetic-related HO, such as progressive ossifying fibrodysplasia (FOP), which is a rare systemic progressive connective tissue disease closely related to genetics, and acquired HO, and its common causes include trauma, surgery, central nervous system injury, and burns, which is the most common type in clinical practice [19]. NSAIDs prevented the synthesis of prostaglandin by inhibiting cytase and the differentiation of ectopic ossification progenitor cells into the osteoblasts, thereby preventing HO formation [20]. It is the most commonly used method to prevent HO in clinical practice; however, it caused gastrointestinal injury and postpone fracture healing. Moreover, the effect of the preventive treatment of HO is not stable; thus, the use of this drug has some limitations [21].

Radiotherapy is a classical method of preventing HO. Direct irradiation of the site prone to HO interferes with osteogenic progenitor cells and their survival microenvironment, thus cutting off the pathway of HO formation [22]. Some studies have compared the efficacy of radiotherapy and NSAIDs and found no significant difference between the two [23]. Although radiotherapy is still the conventional method of preventing HO, radiotherapy may induce serious systemic complications; thus, given the inconvenience and high treatment cost, it has been gradually replaced by drug therapy [24].

Researchers have found that retinoic acid receptor (RAR) activators can effectively inhibit HO and have no significant effects on fracture and wound healing. Shimono et al. found that RAR-*γ* activators CD1530 and NRX195183 can significantly inhibit heterotopic bone formation in HO rats. Meanwhile, RAR-*γ* activator inhibited chondrogenesis, reduced the number of blood vessels and osteoclasts in rats, and significantly downregulated SOX9 and RUNX2 [25].

The local tissue microenvironment is also one of the influencing factors of HO formation [26]. In the early stage of the trauma, the inflammatory factor level was increased as the acute phase reaction and induced local inflammation and tissue repair. At the same time, the signal level of related signal transduction pathway increased. TGF-*β* and other osteogenic inducible factors also increased, which cause ossification progenitor cell proliferation and differentiation and finally stimulate the occurrence of HO. [27].

The role of the inflammatory microenvironment in HO formation has received increasing attention. In the early stage after inflammation and traumatic stimulation, an acute reaction increases the levels of inflammatory factors such as IL-6 and TNF-*α* at the injury site and induces local inflammatory response and tissue repair [28]. Meanwhile, signal levels of related signal transduction pathways and osteogenic and chondrogenic induction factors such as OCN, RUNX2, and SOX9 were increased [29]. Dighe et al. found that the inflammatory microenvironment formed by IFN-*γ* and T cells can increase the osteogenic activity of MSCs [30]. Wang et al. also confirmed that high levels of Smad in an inflammatory environment induced the recruitment of several MSCs, leading to HO [31]. Bressan et al. found that the inflammatory microenvironment induced by Ti particles would lead to the production of high reactive oxygen species (ROS) levels, recruiting abnormal quantity of neutrophils to produce high level of metalloproteinase and the degradation of collagen fibers, thus destroying the balance of bone regeneration [32]. Evans et al. found that inflammatory factor IL-3 was elevated in patients with combat wounds [33]. Similarly, Schett also found that cytokines such as IL-6, IL-10, and MCP-1 were elevated in patients with gunshot wounds. Inflammatory cytokines IL-3, IL-6, IL-10, and MCP-1 are often considered as markers of inflammatory microenvironment [34]. Spinal cord injuries and traumatic brain injuries can also result in development of HO [35], Macrophages may secrete oncostatin M to mediate inflammation when spinal cord injury occurs [36].

Studies have found that the inflammatory microenvironment caused by external stimuli such as trauma or hypoxia can cause a series of changes in cell homeostasis, such as the occurrence of metabolic acidosis and extracellular pH changes [37]. Acidification of the cellular microenvironment is found in inflammation pathological states. It can affect cell function and phenotype and aggravate the pathological process [38]. In bone sarcomas, acidic pH environment increases stemness, invasion, angiogenesis, metastasis, and resistance to therapy of cancer cells [39]. Therefore, it is necessary to observe the changes of inflammatory microenvironment, which can effectively indicate the progression of HO.

Bone morphogenetic protein (BMP), a substance found in the extracellular matrix of the bone, is an activator of the Smad signaling pathway and can induce HO in soft tissues [40]. The activated Smad signal transduction pathway plays a central role in bone formation, bone repair, and HO. In HO animal models, the Smad signal transduction pathway was upregulated horizontally. BMP phosphorylates Smad1 and Smad5 activates BMP I receptors (such as ALK2) and BMP II receptors to phosphorylation. The phosphorylated Smad1 and Smad5 combined with Smad4 formed a complex, and the complex enters the nucleus. In the nucleus, these complexes interact with other osteogenic transcription factors, such as RUNX2, and then bind to target genes to regulate osteogenic processes [41].

Osteogenic progenitor cells, osteogenic signal transduction pathways, and local tissue microenvironment are currently the focus of HO pathogenesis research. Based on the experimental results of this study, we will try to answer the mechanism by which palovarotene inhibits HO by acting on the above three elements. Our results demonstrated that TSCs had osteogenic and chondrogenic potential and acted as osteogenic progenitors cells in the course of HO. TSCs underwent osteogenic differentiation in an inflammatory microenvironment dominated by polarized macrophages. Alizarine red and alkaline phosphatase staining presented that, compared with the blank control group, TSCs underwent osteogenic differentiation in an inflammatory microenvironment dominated by polarized macrophages. Under the inflammatory microenvironment, TSC bone deposition and phosphatase levels increased, and immunofluorescence revealed that the Smad and NF-*κ*B signaling pathways were activated, which ultimately induced HO. After palovarotene treatment, OCN, RUNX2, SOX9, TNF-*α*, IL-1*β*, MMP9, and IFN-*γ* were significantly downregulated in HO animal models and cells with inflammatory activation symptoms. Inflammatory cytokines TNF-*α* and IL-1*β* are thought to promote the production of inflammatory mediators. MMP9 and IFN-*γ* are also inflammatory regulators and play a regulatory role in the pathogenesis of HO. OCN and RUNX2 are commonly used as markers of osteogenesis, while SOX9 is often considered a marker of chondrogenesis. In subsequent experiments, we found that molecules such as Id1, Smad5, and P65 were also significantly downregulated in HO animal models with inflammatory activation symptoms after palovarotene administration and in HO cells. As a key molecule of the Smad signaling pathway, Smad5 often indicates the degree of activation of the Smad signaling pathway [9]. As a downstream transcription molecule of the Smad signaling pathway, Id1 also represents the degree of activation of the Smad signaling pathway [42]. In addition, P65 is a key molecule of the NF-*κ*B signaling pathway. The level of P65 and its phosphorylation increased in the Smad-overexpressed cell model, while it decreased in the Smad knockdown cell model, suggesting the codirectional interaction between the Smad and NF-*κ*B signaling pathways [43]. Palovarotene, as a RAR activator, interacts with Smad molecules through the nuclear entry and blocks the activation of Smad and NF-*κ*B signaling pathways, thereby inhibiting HO progression.

## 5. Conclusions

We verified that tendon stem cells (TSCs) activate the Smad signaling pathway and enhance osteogenic differentiation to induce HO under inflammatory microenvironment and trauma stimulation. The NF-*κ*B signaling pathway acts synergistically in this process. Palovarotene may act on the common downstream transcription target of Smad and NF-*κ*B. Palovarotene inhibits HO by blocking Smad and NF-*κ*B signaling pathways, suggesting that palovarotene may be a novel HO inhibitor, while other drugs or antibodies targeting Smad and NF-*κ*B signaling pathways may prevent or treat HO. The expressions of Smad5, Id1, P65, and other proteins may predict HO formation. The above findings suggest new ideas for further study on HO formation and development and provide new methods for its prevention and treatment.

## Figures and Tables

**Table 1:** The primer sequences used in this study.

Gene	Primers
OCN mouse (F)	GGACCATCTTTCTGCTCACTCTGC
OCN mouse (R)	TGTTCACTACCTTATTGCCCTCCTG
RUNX2 mouse (F)	GACTGTGGTTACCGTCATGGC
RUNX2 mouse (R)	ACTTGGTTTTTCATAACAGCGGA
SOX-9-mouse	CGGAACAGACTCACATCTCTCC
SOX-9-mouse	GCTTGCACGTCGGTTTTGG
MMP9 mouse (F)	GGACCCGAAGCGGACATTG
MMP9 mouse (R)	CGTCGTCGAAATGGGCATCT
smad5-mouse-F	GAGCCATCACGAGCTAAAACC
smad5-mouse-R	ACTGGAGGTAAGACTGGACTCT
ID1-mouse-F	CTTCCCCAACCACCAGTACAA
ID1-mouse-R	CTGTGCGAAGGACGAAGAC
TNF-*α*-M-F	GGAACACGTCGTGGGATAATG
TNF-*α*-M-R	GGCAGACTTTGGATGCTTCTT
IL-1*β*-M-F	AGCATCCAGCTTCAAATC
IL-1*β*-M-R	ATCTCGGAGCCTGTAGTG
IFN-*γ*-M-F	AGTGGCATAGATGTGGAA
IFN-*γ*-M-R	CTCAAACTTGGCAATACTC
OCN rat (F)	ATGAACTAAAGCCTCTGGAAT
OCN rat (R)	GGTTGTACTCGCTGTGCC
SOX-9-rat-F	GCACATCAAGACGGAGCAA
SOX-9-rat-R	AGGTGAAGGTGGAGTAGAGCC
smad5-rat-F	AGCCTGTTGCCTATGAAG
smad5-rat-R	TGGTTGAGTTGCGATTAA
ID1-rat-F	GTGGTGCTTGGTCTGTCGG
ID1-rat-R	GGTTCTGAGGCAGGGTAGGC
smad1-mouse-F	GCTTCGTGAAGGGTTGGGG
smad1-mouse-R	CGGATGAAATAGGATTGTGGGG

## Data Availability

All data included in this study are available upon request by contact with the corresponding author.

## Abbreviations

HO: Heterotopic ossification
FOP: Progressive ossifying fibrodysplasia
TSCs: Tendon stem cells
RAR: Retinoic acid receptor
NF-*κ*B: Nuclear factor-kappa B
NSAIDs: Nonsteroidal anti-inflammatory drugs
MSCs: Mesenchymal stem cells
ARS: Alizarin red staining
ALP: Alkaline phosphatase
RT-PCR: Reverse transcription-polymerase chain reaction
DMSO: Dimethyl sulfoxide
EDTA: Ethylenediamine tetraacetic acid
IM: Inflammatory microenvironment
HE: Hematoxylin-eosin staining
SOFG: Safranin O-fast green
BMP: Bone morphogenetic protein
OCN: Osteocalcin
RUNX2: Runt-related transcription factor 2
SOX9: Sry-related HMG box-9
ROS: Reactive oxygen species.

## Supplementary Materials

Supplementary Materials Supplementary Figure 1: original full gels images of Western blots.

## References

[1] Xu R., Hu J., Zhou X., Yang Y. (2018). Heterotopic ossification: mechanistic insights and clinical challenges. *Bone*.

[2] Meyers C., Lisiecki J., Miller S. (2019). Heterotopic ossification: a comprehensive review. *JBMR Plus*.

[3] Kaplan F. S., Shore E. M. (2011). Derailing heterotopic ossification and RARing to go. *Nature Medicine*.

[4] Shimono K., Morrison T. N., Tung W. (2010). Inhibition of ectopic bone formation by a selective retinoic acid receptor *α*-agonist: a new therapy for heterotopic ossification?. *Journal of Orthopaedic Research*.

[5] O'Brien E. J. O., Frank C. B., Shrive N. G., Hallgrímsson B., D. Hart A. (2012). Heterotopic mineralization (ossification or calcification) in tendinopathy or following surgical tendon trauma. *International Journal of Experimental Pathology*.

[6] Kaplan F. S., Glaser D. L., Hebela N., Shore E. M. (2004). Heterotopic ossification. *The Journal of the American Academy of Orthopaedic Surgeons*.

[7] Yang L., Tang C., Chen Y. (2019). Pharmacological inhibition of Rac1 activity prevents pathological calcification and enhances tendon Regeneration. *ACS Biomaterials Science & Engineering*.

[8] Chen Y., Xie Y., Liu M. (2018). Controlled-release curcumin attenuates progression of tendon ectopic calcification by regulating the differentiation of tendon stem/progenitor cells. *Materials Science and Engineering: C*.

[9] Tirone M., Giovenzana A., Vallone A. (2019). Severe heterotopic ossification in the skeletal muscle and endothelial cells recruitment to Chondrogenesis are enhanced by monocyte/macrophage depletion. *Frontiers in Immunology*.

[10] Liu Y., Zou X., Chai Y., Yao Y. M. (2014). Macrophage polarization in inflammatory Diseases. *International Journal of Biological Sciences*.

[11] Hanisch M., Hanisch L., Fröhlich L. F., Werkmeister R., Bohner L., Kleinheinz J. (2018). Myositis ossificans traumatica of the masticatory muscles: etiology, diagnosis and treatment. *Head & Face Medicine*.

[12] Lees-Shepard J. B., Nicholas S. E., Stoessel S. J. (2018). Palovarotene reduces heterotopic ossification in juvenile FOP mice but exhibits pronounced skeletal toxicity. *eLife*.

[13] Cappato S., Gamberale R., Bocciardi R., Brunelli S. (2020). Genetic and acquired heterotopic ossification: a translational tale of mice and men. *Biomedicines*.

[14] Ju J., Yu D., Xue F. (2019). Inhibition of Nf-κb prevents trauma-induced heterotopic ossification in rat model. *Connective Tissue Research*.

[15] Chen S., Guttridge D. C., Tang E., Shi S., Guan K. L., Wang C. Y. (2001). Suppression of tumor necrosis factor-mediated apoptosis by nuclear factor kappa B-independent bone morphogenetic protein/Smad signaling. *The Journal of Biological Chemistry*.

[16] Harvey T., Flamenco S., Fan C. M. (2019). A Tppp3+Pdgfra+ tendon stem cell population contributes to regeneration and reveals a shared role for PDGF signalling in regeneration and fibrosis. *Nature Cell Biology*.

[17] Xuan W., Qu Q., Zheng B., Xiong S., Fan G. H. (2015). The chemotaxis of M1 and M2 macrophages is regulated by different chemokines. *Journal of Leukocyte Biology*.

[18] Pavey G. J., Qureshi A. T., Tomasino A. M. (2016). Targeted stimulation of retinoic acid receptor-*γ* mitigates the formation of heterotopic ossification in an established blast-related traumatic injury model. *Bone*.

[19] Dey D., Wheatley B. M., Cholok D. (2017). The traumatic bone: trauma-induced heterotopic ossification. *Translational Research*.

[20] Łęgosz P., Drela K., Pulik Ł., Sarzyńska S., Małdyk P. (2018). Challenges of heterotopic ossification—molecular background and current treatment strategies. *Clinical and Experimental Pharmacology & Physiology*.

[21] Burd T. A., Hughes M. S., Anglen J. O. (2003). Heterotopic ossification prophylaxis with indomethacin increases the risk of long-bone nonunion. *The Journal of Bone and Joint Surgery. British Volume*.

[22] Coventry M. B., Scanlon P. W. (1981). The use of radiation to discourage ectopic bone. A nine-year study in surgery about the hip. *The Journal of Bone and Joint Surgery. American Volume*.

[23] Vavken P., Castellani L., Sculco T. P. (2009). Prophylaxis of heterotopic ossification of the hip: systematic review and meta-analysis. *Clinical Orthopaedics and Related Research*.

[24] Bastepe M. (2018). GNAS mutations and heterotopic ossification. *Bone*.

[25] Shimono K., Tung W., Macolino C. (2011). Potent inhibition of heterotopic ossification by nuclear retinoic acid receptor-*γ* agonists. *Nature Medicine*.

[26] Adamopoulos I. E. (2018). Inflammation in bone physiology and pathology. *Current Opinion in Rheumatology*.

[27] Zhang Q., Zhou D., Wang H., Tan J. (2020). Heterotopic ossification of tendon and ligament. *Journal of Cellular and Molecular Medicine*.

[28] Juarez J. K., Wenke J. C., Rivera J. C. (2018). Treatments and preventative measures for trauma-induced heterotopic ossification: a review. *Clinical and Translational Science*.

[29] Zhong B., Zhang C., Guo S., Zhang C. (2017). Rational design of cyclic peptides to disrupt TGF-*Β*/SMAD7 signaling in heterotopic ossification. *Journal of Molecular Graphics & Modelling*.

[30] Dighe A. S., Yang S., Madhu V., Balian G., Cui Q. (2013). Interferon gamma and T cells inhibit osteogenesis induced by allogeneic mesenchymal stromal cells. *Journal of Orthopaedic Research*.

[31] Wang X., Li F., Xie L. (2018). Inhibition of overactive TGF-*β* attenuates progression of heterotopic ossification in mice. *Nature Communications*.

[32] Bressan E., Ferroni L., Gardin C. (2019). Metal nanoparticles released from dental implant surfaces: potential contribution to chronic inflammation and peri-implant bone loss. *Materials*.

[33] Evans K. N., Forsberg J. A., Potter B. K. (2012). Inflammatory cytokine and chemokine expression is associated with heterotopic ossification in high-energy penetrating war injuries. *Journal of Orthopaedic Trauma*.

[34] Schett G. (2011). Effects of inflammatory and anti-inflammatory cytokines on the bone. *European Journal of Clinical Investigation*.

[35] Sullivan M. P., Torres S. J., Mehta S., Ahn J. (2013). Heterotopic ossification after central nervous system trauma: a current review. *Bone & Joint Research*.

[36] Alexander K. A., Tseng H. W., Fleming W. (2019). Inhibition of JAK1/2 tyrosine kinases reduces neurogenic heterotopic ossification after spinal cord injury. *Frontiers in Immunology*.

[37] Matsuo K., Chavez R. D., Barruet E., Hsiao E. C. (2019). Inflammation in fibrodysplasia ossificans progressiva and other forms of heterotopic ossification. *Current Osteoporosis Reports*.

[38] Riemann A., Reime S., Gießelmann M., Thews O. (2020). Extracellular acidosis regulates the expression of inflammatory mediators in rat epithelial cells. *Advances in Experimental Medicine and Biology*.

[39] Di Pompo G., Cortini M., Baldini N., Avnet S. (2021). Acid microenvironment in bone sarcomas. *Cancers*.

[40] Wu M., Chen G., Li Y.-P. (2016). TGF-*β* and BMP signaling in osteoblast, skeletal development, and bone formation, homeostasis and disease. *Bone Research*.

[41] Lin H., Shi F., Gao J., Hua P. (2019). The role of Activin a in fibrodysplasia ossificans progressiva: a prominent mediator. *Bioscience Reports*.

[42] Kitoh H. (2020). Clinical aspects and current therapeutic approaches for FOP. *Biomedicine*.

[43] Hayden M. S., Ghosh S. (2008). Shared principles in NF-*κ*B signaling. *Cell*.

