# Supplementary material for: Palovarotene Can Attenuate Heterotopic Ossification Induced by Tendon Stem Cells by Downregulating the Synergistic Effects of Smad and NF-κB Signaling Pathway following Stimulation of the Inflammatory Microenvironment
Source: Stem Cells Int. 2022 Apr 28;2022:1560943. doi: 10.1155/2022/1560943 (PMC9071930; doi:10.1155/2022/1560943)
Supplement: Supplementary Materials — Supplementary Figure 1: original full gels images of Western blots. [file 1560943.f1.pdf]

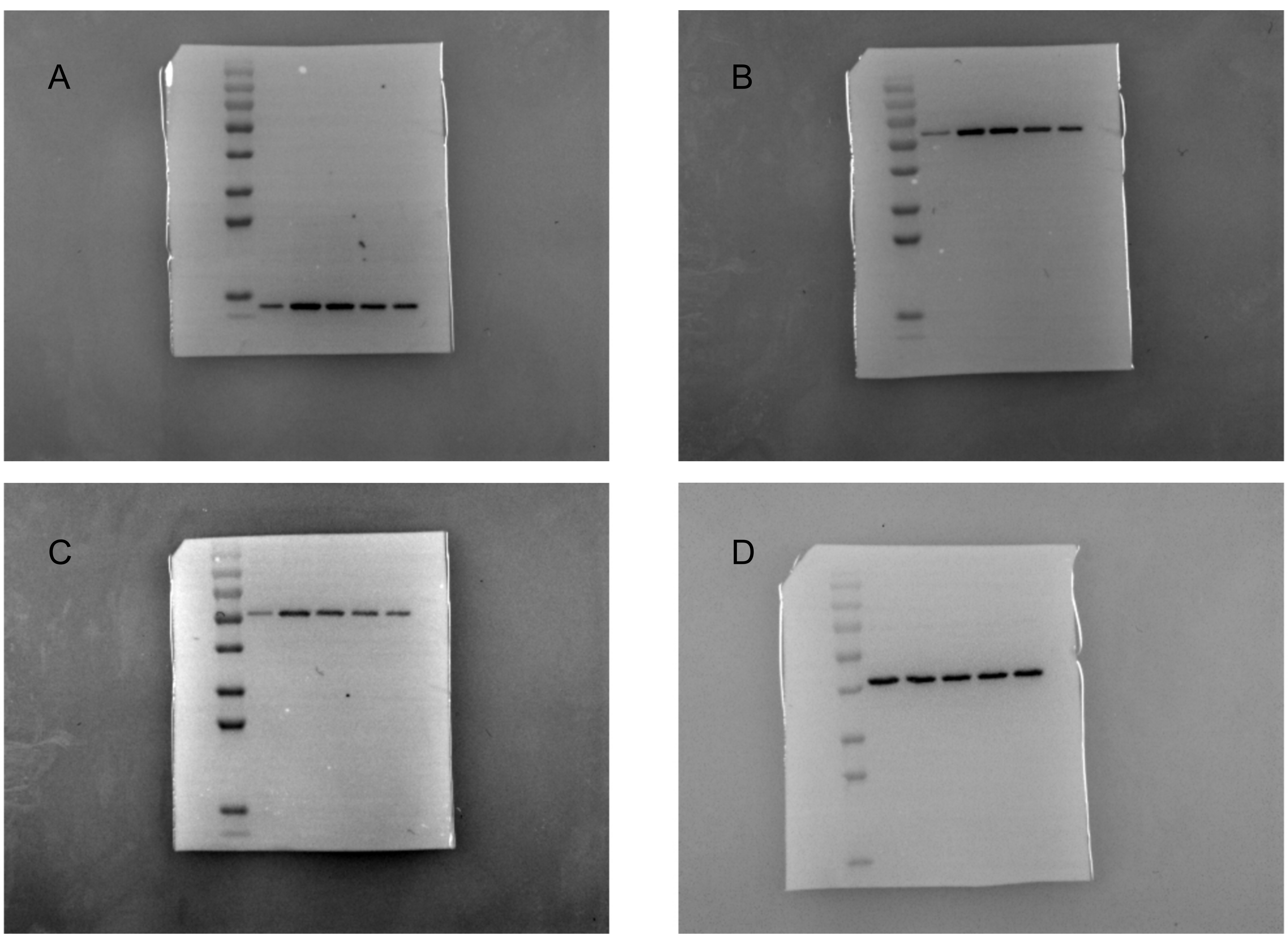

Original full gels pictures of WB in Figure 2C of the manuscript.
(A) OCN
(B) RUNX2
(C) SOX9
(D) β-actin

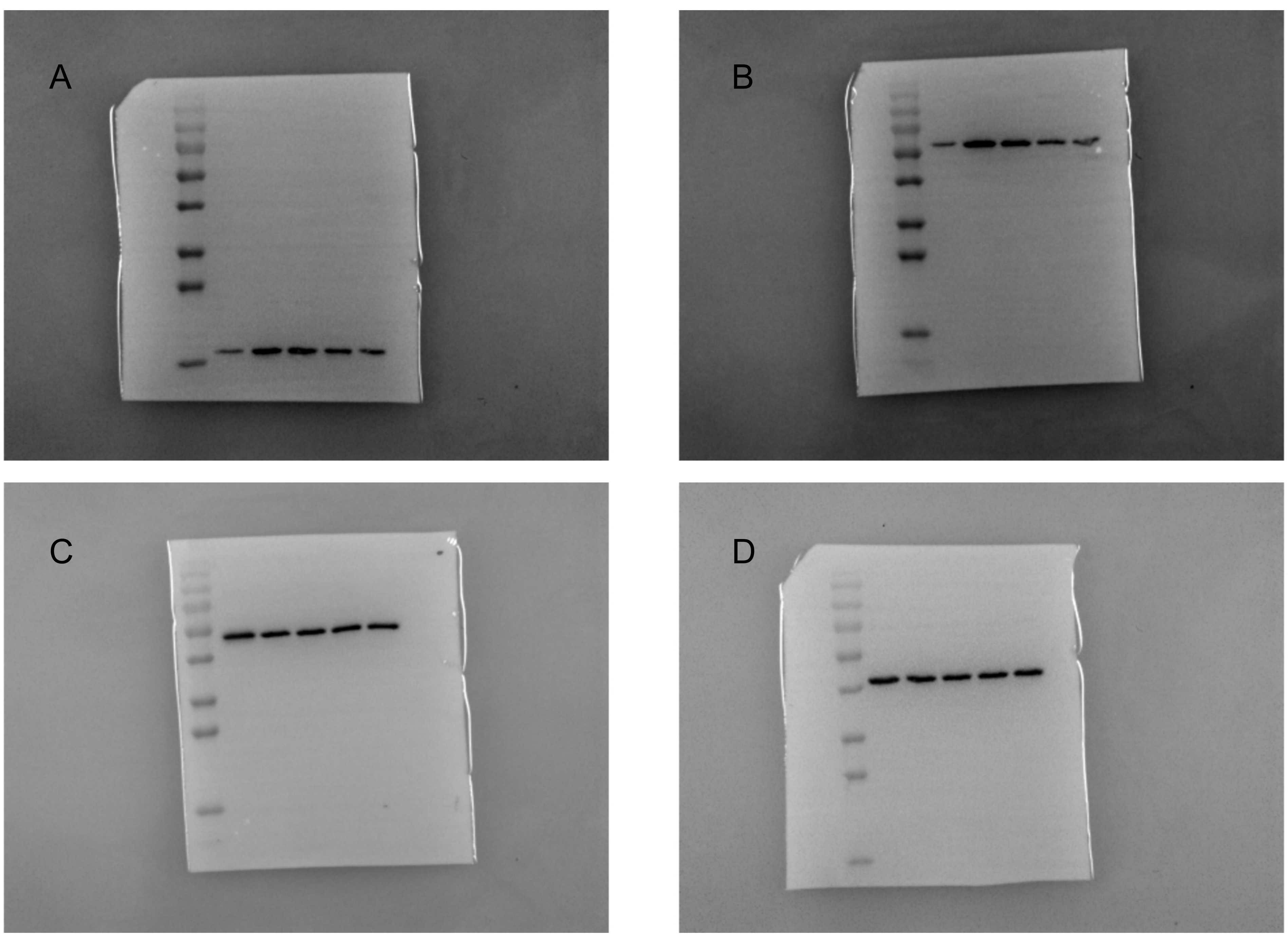

Original full gels pictures of WB in Figure 3A of the manuscript.
(A) ID1
(B) p-Smad5
(C) Smad5
(D) β-actin

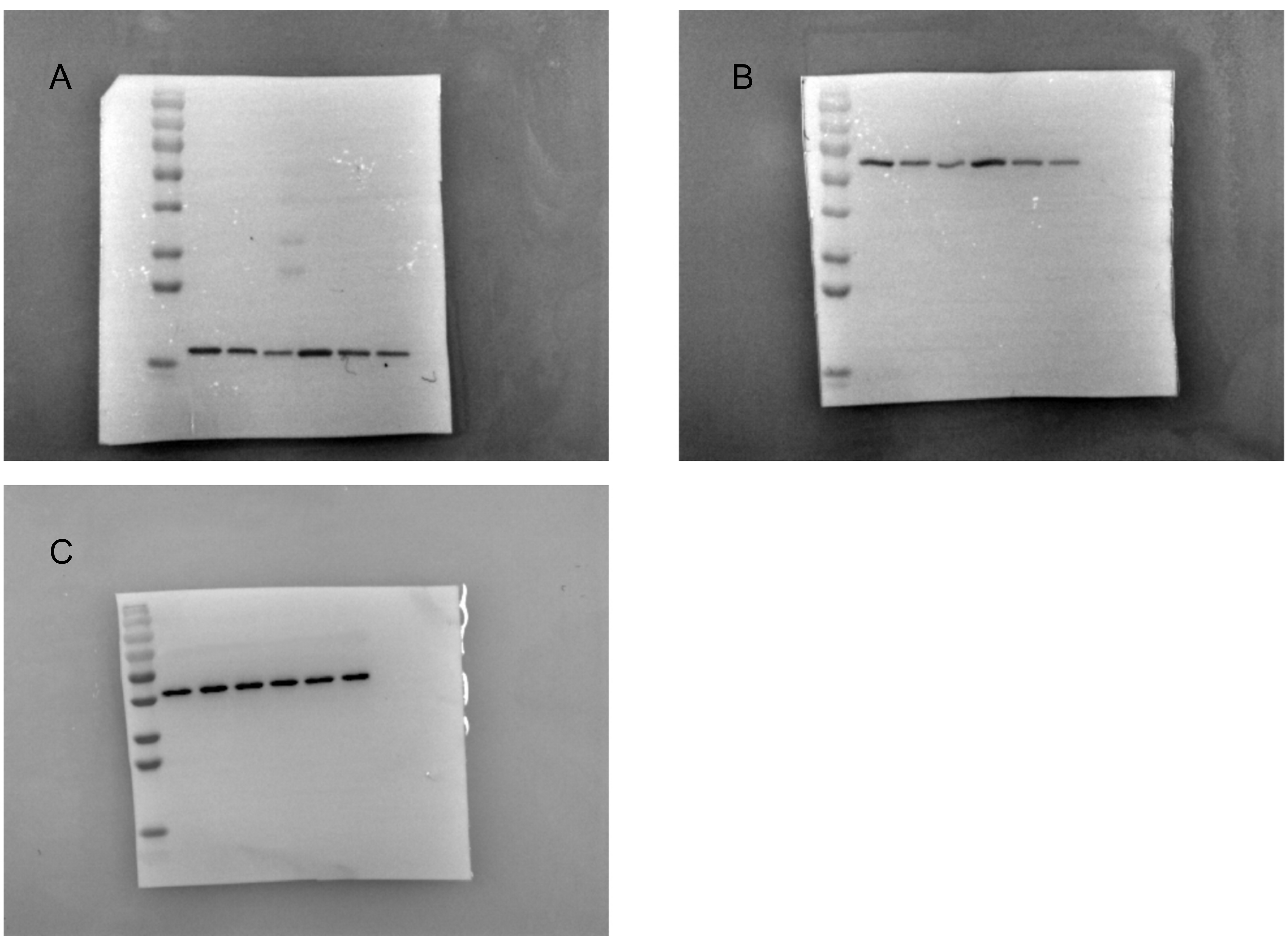

Original full gels pictures of WB in Figure 4C of the manuscript.
(A) ID1
(B) p-P65
(C) β-actin

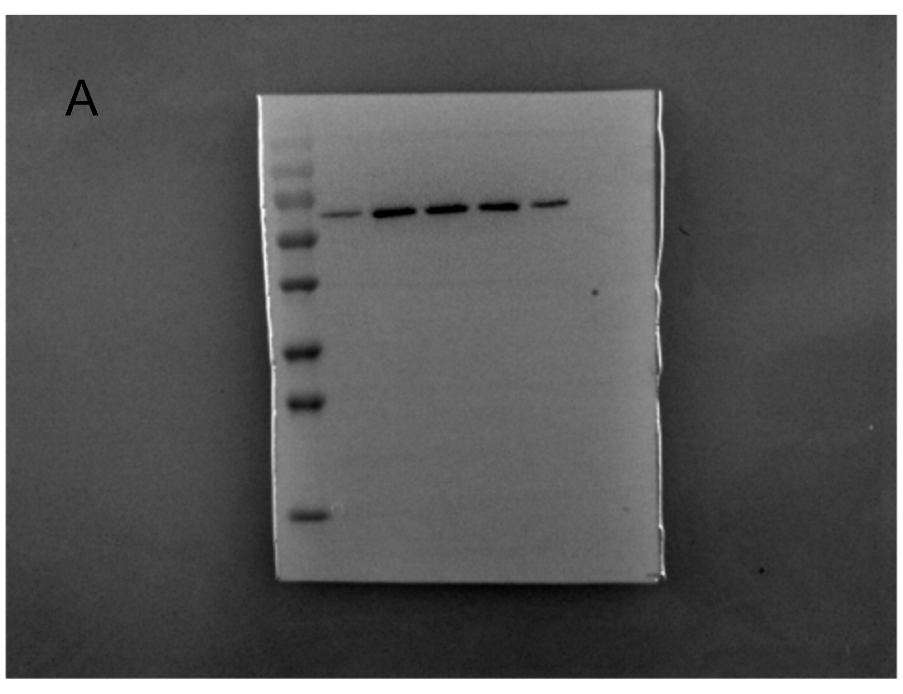

Original full gels pictures of WB in Figure 4E of the manuscript.
(A) P65

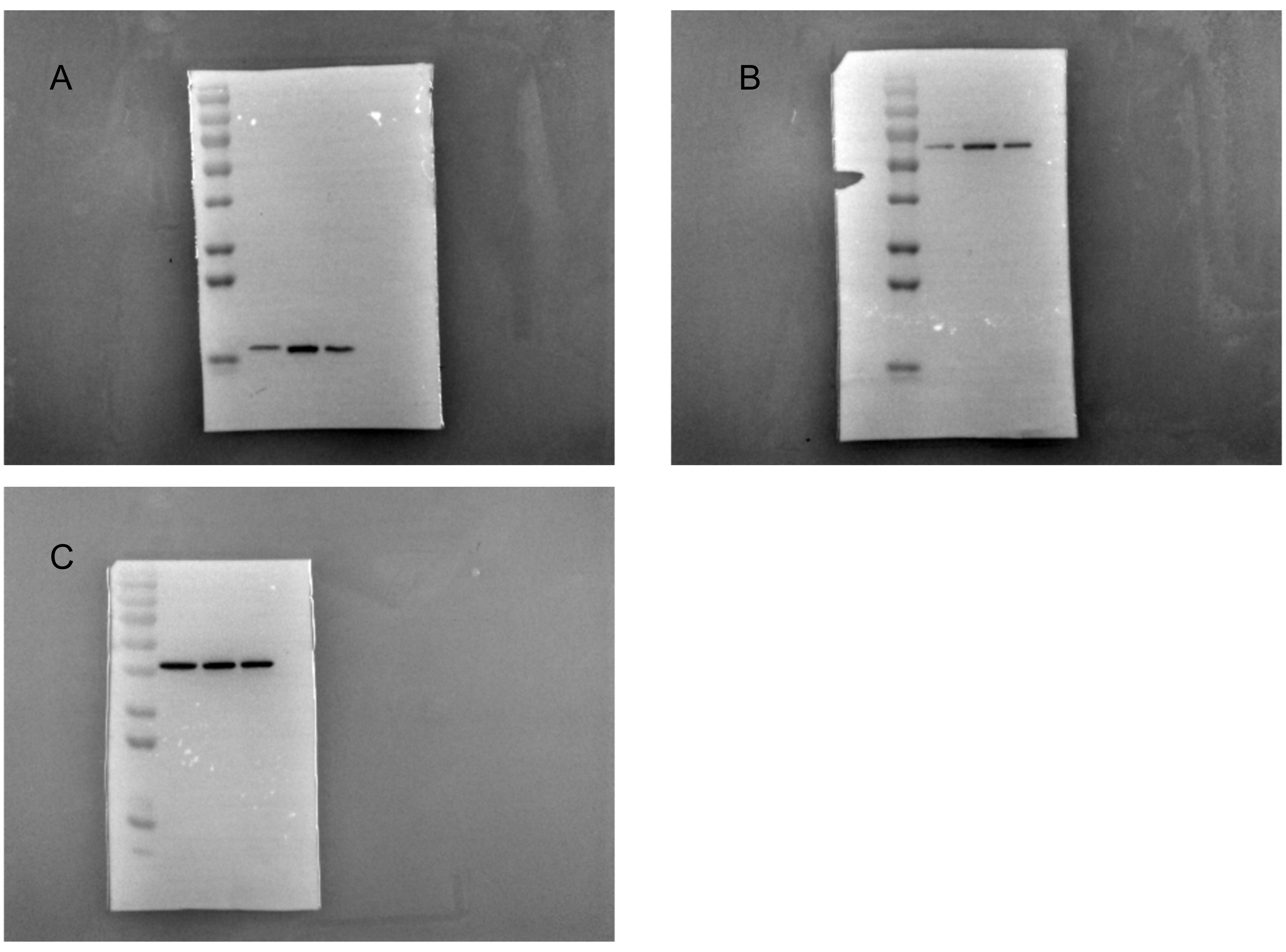

Original full gels pictures of WB in Figure 6D of the manuscript.
(A) ID1
(B) P65
(C) β-actin
